# Renal Ischemia/Reperfusion Injury in Diabetic Rats: The Role of Local Ischemic Preconditioning

**DOI:** 10.1155/2016/8580475

**Published:** 2016-01-26

**Authors:** Sule Ozbilgin, Sevda Ozkardesler, Mert Akan, Nilay Boztas, Mucahit Ozbilgin, Bekir Ugur Ergur, Serhan Derici, Mehmet Ensari Guneli, Reci Meseri

**Affiliations:** ^1^Department of Anesthesiology and Reanimation, School of Medicine, Dokuz Eylul University, Izmir, Turkey; ^2^Department of General Surgery, School of Medicine, Dokuz Eylul University, Izmir, Turkey; ^3^Department of Histology and Embryology, School of Medicine, Dokuz Eylul University, Izmir, Turkey; ^4^Department of Experienced Laboratory Animal Science, School of Medicine, Dokuz Eylul University, Izmir, Turkey; ^5^Department of Nutrition and Dietetics, Izmir Ataturk School of Health, Ege University, Izmir, Turkey

## Abstract

*Background*. The aim of this study was to evaluate the effects of local ischemic preconditioning using biochemical markers and histopathologically in the diabetic rat renal IR injury model.* Methods*. DM was induced using streptozotocin. Rats were divided into four groups: Group I, nondiabetic sham group (*n* = 7), Group II, diabetic sham group (*n* = 6), Group III, diabetic IR group (diabetic IR group, *n* = 6), and Group IV, diabetic IR + local ischemic preconditioning group (diabetic IR + LIPC group, *n* = 6). Ischemic renal injury was induced by clamping the bilateral renal artery for 45 min. 4 h following ischemia, clearance protocols were applied to assess biochemical markers and histopathologically in rat kidneys.* Results*. The histomorphologic total cell injury scores of the nondiabetic sham group were significantly lower than diabetic sham, diabetic IR, and diabetic IR + LIPC groups. Diabetic IR group scores were not significantly different than the diabetic sham group. But diabetic IR + LIPC group scores were significantly higher than the diabetic sham and diabetic IR groups.* Conclusion*. Local ischemic preconditioning does not reduce the risk of renal injury induced by ischemia/reperfusion in diabetic rat model.

## 1. Introduction

Diabetes mellitus (DM) is a common and increasing chronic metabolic disease progressing with hyperglycemia, dyslipidemia, glycosuria, and metabolic disorders [1, 2]. Diabetic nephropathy is a cause of end-stage renal failure [3]. Diabetes mellitus is defined as a risk factor for the development of acute renal damage in a variety of clinical situations such as radio-contrast nephropathy or after cardiopulmonary bypass [4, 5].

Clinical studies of diabetes mellitus patients have previously reported increased susceptibility of the kidney to acute kidney injury (AKI). Studies of experimental models of this disease have shown that diabetic rats have increased susceptibility to renal ischemia/reperfusion injury (IRI) [6–8]. Various methods have been developed to prevent ischemia-reperfusion (IR) injury. One of these methods is local ischemic preconditioning (LIPC) which was described for the heart by Murry et al. in 1986. In local ischemia preconditioning the aim is to expose the organ to short IR periods before the ischemia duration to protect from IR damage. However this method has disadvantages such as causing trauma to large veins and stress to organs [7]. Another method is remote ischemic preconditioning (RIPC). Increasing the resistance of an organ to ischemia with the application of short ischemia-reperfusion episodes to another organ is called RIPC [8]. Thus the target organ is not exposed to direct stress [8]. For the treatment of renal IRI, supportive therapy remains, and therefore improved treatment strategies to prevent renal IR injury are drawing attention from scholars [7].

Renal IRI may be shown histomorphologically and biochemically. Both inflammation and apoptosis coexist in renal IR injury. During hypoxia, caspase activity increases as a result of intracellular Ca^2+^ accumulation. Caspase becomes activated in ischemic tissues and is an indicator of cell death [7, 9]. These changes, which can be observed in tubular cells, may cause the loss of brush borders of proximal tubular cells and spill out from the basement membrane of the cells into the tubular lumen, with eventual tubule obstruction. Biochemically, in addition to BUN, creatinine, and GFR, the new marker of NGAL has entered the agenda recently. Neutrophil gelatinase-associated lipocalin is shown to be the most expressed protein in the kidney a short time before creatinine after acute ischemic and nephrotoxic injury. Renal function can be monitored using the neutrophil gelatinase-associated lipocalin (NGAL) biomarker. After acute kidney injury NGAL protein is easily identified in blood and urine. In fact, NGAL is one of the earliest markers of renal injury after ischemia or nephrotoxic injury in animal models [10]. Most of the studies pertaining to the use of NGAL have come from cardiothoracic surgery, transplant, and critical care literature. Based on some of these findings, it has been noted that increased levels of serum NGAL may be seen as soon as two hours after a renal insult [10]. It seems that urine and plasma levels of NGAL are both equally acceptable as markers of acute kidney injury (AKI) [11], although there are some reports that urinary NGAL may have a slightly higher predictive value, at least in the critical care environment.

The aim of this study was to evaluate the effects of local ischemic preconditioning using biochemical markers (BUN, Cr, and NGAL) and histopathologically in the diabetic rat renal IR injury model.

## 2. Methods

After obtaining permission from the Dokuz Eylul University School of Medicine (DEUSM) Local Animal Experiments Ethical Committee (Date: 03.09.2014, protocol number: 27/2014, Yılmaz O), the research was carried out at the DEUSM Animal Experiment Laboratories. Twenty-eight adult Wistar albino rats weighing 230–300 g were used in this study. The animals were housed in a light controlled room with a 12 h light/dark cycle and allowed access to food and water. Experimental protocols and animal care methods in the experiment were approved by the Experimental Animal Research Committee of our institution.

### 2.1. Induction of Diabetes

STZ was used to induce diabetes as described previously [12]. To induce the diabetes model, 45 mg/kg streptozotocin (STZ) (STZ, Sigma Chemical Co., St. Louis, MO, USA) was administered intraperitoneally in a single dose. STZ was prepared in a 0.1 M phosphate-citrate buffer (pH: 4.5) and an equal volume of buffer was injected ip into the control sham group without induced diabetes. STZ was prepared freshly and used immediately. Three days after this application a blood sample was taken from the tail. Rats with blood sugar > 250 mg/dL on glycometry of the sample were accepted as diabetic. The rats were monitored for one month in the experimental animals laboratory and then the study began. Within this time, weight changes and blood glucose measurements were recorded.

### 2.2. Study Design

Rats were divided into four groups:* Group I*, nondiabetic sham group (*n* = 7),* Group II*, diabetic sham group (*n* = 7),* Group III*, diabetic IR group (diabetic IR group, *n* = 7), and* Group IV*, diabetic IR + local ischemic preconditioning group (diabetic IR + LIPC group, *n* = 7).

The rats were anesthetized with ketamine (50 mg/kg ip) and xylazine hydrochloride (10 mg/kg ip) and bilateral renal pedicles were exposed after laparotomy. After anesthesia, the rats were heated with a heating lamp to maintain a rectal body temperature of 37°C. At the beginning of the study, 1 mL of blood sample was drawn from the lateral tail vein for the measurement of basal blood glucose before abdominal incision. For IRI induction, bilateral renal pedicle occlusion was performed with hemostasis atraumatic microvascular clamp for 45 min. At the end of the ischemic period, the clips were removed to allow blood reperfusion. In nondiabetic sham and also diabetic sham group, bilateral renal pedicles were exposed without any intervention after laparotomy. In diabetic IR group, after 45 min of ischemia, the bilateral atraumatic microvascular clamp was removed and reperfusion of the kidneys was allowed to continue for 4 hours. In diabetic IR + LIPC group, bilateral renal pedicles were exposed. Left renal pedicles were maintained with atraumatic microvascular clamp compression. Four cycles of 4 min of ischemia were performed followed by 11 min of reperfusion for the left kidney (65 minutes total). For total renal ischemia, after 5 min the bilateral renal pedicles were clamped for 45 min and then these clamps were removed and reperfusion of the kidneys was allowed for 4 hours.

To protect the rats from hypothermia, the operating table was heated with a lamp heater throughout the study and rectal body temperature was measured with a probe and maintained at 37–37.5°C. To avoid dehydration and hypothermia 3 mL/kg/h subcutaneous isotonic fluid solution was administered during the operation. During the waiting time, the abdomen was closed with a moist sterile pad and surgical forceps. At the end of reperfusion, the animals were anesthetized, blood samples were drawn from the right atrium for the measurement of renal function parameters, and left kidneys were excised. The kidneys were fixed in 10% buffered formalin and embedded in paraffin wax, cut to 4-5 *μ*m, and stained with hematoxylin and eosin for histological studies using a light microscope.

### 2.3. Exclusion Criteria

Rats in need of resuscitation were excluded from the study.

### 2.4. Renal Ischemia-Reperfusion Model

The right and left renal pedicles were exposed after laparotomy. Total renal ischemia was maintained by compressing the bilateral renal pedicles with microvascular clips. Adequate occlusion was confirmed by a lack of pulsation in the renal pedicles and presence of pallor in the kidneys. After the ischemic period, the microvascular clips were removed and reperfusion occurred. The cessation of blood flow was confirmed using a laser current meter (Laser Flo BPM2, Vasamedic, USA).

### 2.5. Local Ischemic Preconditioning Model

For local ischemic preconditioning, a method that has been shown to be effective by perfusion scintigraphy and a laser meter was used [13, 14]. For this purpose, bilateral renal pedicles were exposed. Left renal pedicles were maintained with atraumatic microvascular clip compression. Four cycles of 4 min of ischemia were performed followed by 11 min of reperfusion for the left kidney (65 minutes total). For total renal ischemia purposes, after 5 min, the bilateral renal pedicles were clamped for 45 min and then these clips were removed and reperfusion of the kidneys was allowed for 4 hours.

### 2.6. Histomorphological Evaluation of Renal Tissue

Renal tissue sections were evaluated after ischemia-reperfusion using light microscopy by two histologists blinded to the animal groups for structural changes in proximal tubules (tubular atrophy, loss of tubular brush border, vacuolization, tubular dilatation, and cast formation), mononuclear cells (MNCs) infiltration, interstitial structural changes, and renal corpuscle morphology. The cross-sectional images were scored semiquantitatively in terms of tubulointerstitial damage. Scoring was conducted as follows: 0 = not at all, 1 = 0–25%, 2 = 26–45%, 3 = 46–75%, and 4 = 76–100% [15].

### 2.7. Biochemical Evaluation

The blood urea nitrogen, blood creatinine level, and serum NGAL levels were measured 4 hours after reperfusion in Dokuz Eylul University Medical Faculty Hospital Biochemistry Laboratory. Blood urea nitrogen and blood creatinine levels were analyzed photometrically with a* Beckman AU 5800* autoanalyzer. Serum NGAL levels were analyzed with the ELISA method using a Boster trade kit (Boster Biological Technology Co., CA; cat number: EK0855, USA). According to the manufacturer's prospectus, the NGAL detection limit is 10 pg/mL with measurement interval of 78 to 5000.

### 2.8. Statistical Analysis

SPSS 15.0 (Statistical Package for the Social Sciences ver. 15, Chicago, IL, USA) was used. Continuous variables are presented as mean ± SD and median (minimum-maximum). For univariate analysis, Mann-Whitney *U* test was used for comparison of two groups. In order to determine weight and blood glucose level fluctuations over time Friedman repeated measurement was conducted. The level of statistical significance was accepted as *p* < 0.05.

## 3. Results

A total of 28 rats were included in the study. In 21 rats monitored for one month for the diabetes protocol, 2 rats were exitus. One rat in the diabetic IR group died during the ischemia period and was excluded from the study; thus, 25 subjects completed the study.

**Figure 1:**
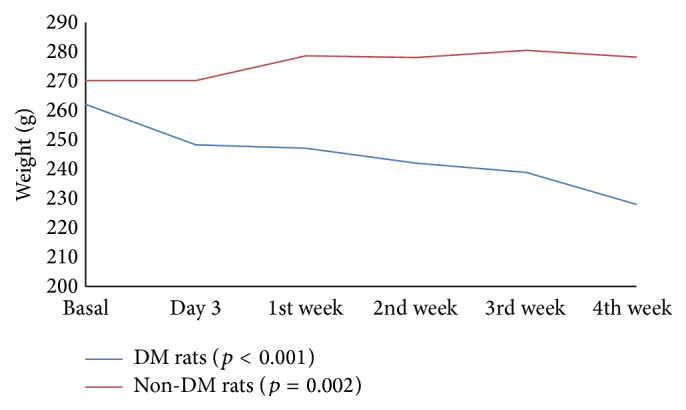
Weight comparison of diabetic and nondiabetic rats. *p* value: Friedman repeated measures.

There was no significant difference between diabetic and nondiabetic rats in terms of basal weight (*p* = 0.060) and glucose values (*p* = 0.611). During the month of monitoring, the weight measurements of diabetic rats significantly decreased over time, while the weight of nondiabetic rats was found to increase during the same period (Figure 1). Body weights were not significantly different between the groups. However, blood glucose values were significantly different among the nondiabetic and diabetic rat groups. Blood glucose levels in nondiabetics rats were significantly lower than those in diabetic rats (all *p* < 0.001) (Table 1). Blood glucose levels were not significantly different between Groups II, III, and IV (*p* > 0.05).

The histopathological scores and biochemical evaluation of the rats in all groups are presented in Tables 2 and 3. The histomorphologic total cell injury scores (presented in Table 1) of the nondiabetic sham group were significantly lower than diabetic sham, diabetic IR, and diabetic IR + LIPC groups (resp., *p* = 0.004, *p* = 0.002, and *p* = 0.002). Diabetic IR group scores were not significantly different to the diabetic sham group (*p* = 0.082). But diabetic IR + LIPC group scores were significantly higher than in the diabetic sham and diabetic IR groups (resp., *p* = 0.003 and *p* = 0.020).

### 3.1. Mononuclear Cell Infiltration

**Figure 2:**
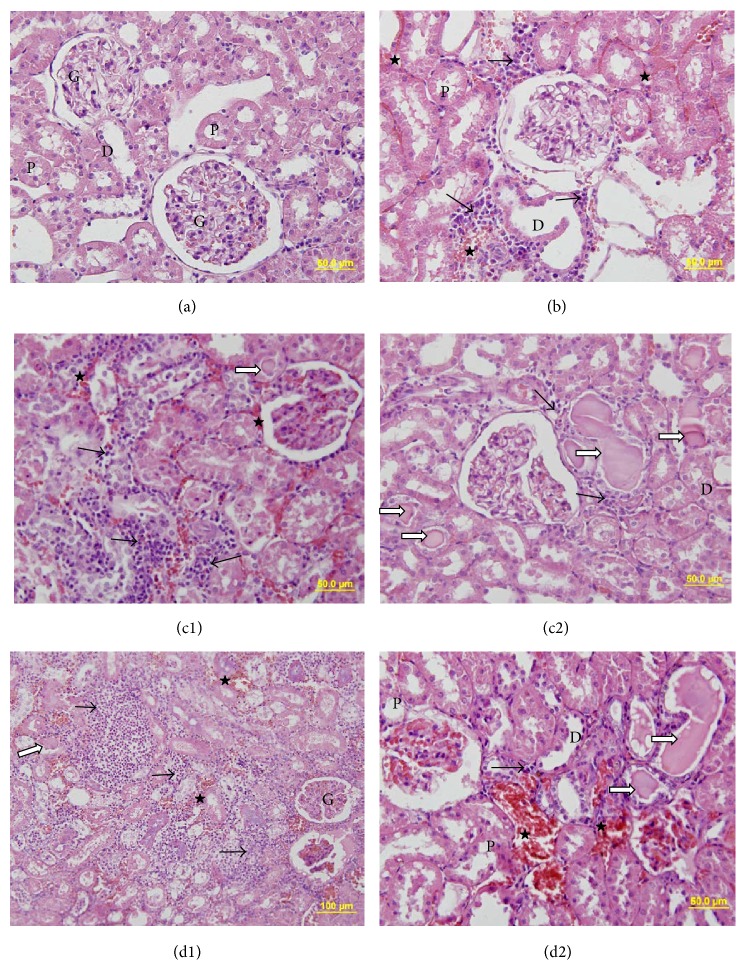
Representative kidney sections belong to (a) nondiabetic sham group, (b) diabetic sham group, (c1 and c2) diabetic ischemia-reperfusion (diabetic IR) group, and (d1 and d2) diabetic IR + LIPC group, respectively. Mononuclear infiltrations marked with black arrow (→), hyalin substance deposition marked with white arrow with black edges (⇒), and erythrocyte extravasations marked with (★). In micrographs proximal tubules, distal tubules and renal glomerulus are shown with (P), (D), and (G), respectively.

The mononuclear cell infiltration scores of the nondiabetic sham group were significantly lower than those of the diabetic sham, diabetic IR, and diabetic IR + LIPC groups (resp., *p* = 0.008, *p* = 0.004, and *p* = 0.003). The difference between the scores of the diabetic sham and diabetic IR (*p* = 0.269) groups was not statistically significant. Also the difference between the scores of the diabetic IR and diabetic IR + LIPC groups was not statistically significant (*p* = 0.206) (Table 2, Figure 2).

The capillary vasodilation of the nondiabetic sham group was statistically significantly lower than that of diabetic sham, diabetic IR, and diabetic IR + LIPC groups (resp., *p* = 0.010, *p* = 0.006, and *p* = 0.001). The scores of the diabetic IR + LIPC group were significantly higher than the diabetic sham and diabetic IR groups (resp., *p* = 0.006 and *p* = 0.043) (Table 2, Figure 2).

### 3.2. Structural Changes in the Proximal Tubules

Structural changes in the proximal tubules of the nondiabetic sham group were significantly lower than those of the diabetic sham, diabetic IR, and diabetic IR + LIPC groups (*p* = 0.003, *p* = 0.001, and *p* = 0.001, resp.). The diabetic IR + LIPC group displayed significantly higher scores than the diabetic sham group (*p* = 0.014) (Table 2, Figure 2).

### 3.3. Biochemical Parameters

There were no significant differences between nondiabetic sham group and the diabetic sham group in the mean values for BUN (*p* = 0.086), creatinine (*p* = 0.567), and NGAL (*p* = 0.153). In the diabetic IR group, the BUN, Cr, and NGAL levels were higher than in the diabetic sham group, though this was not statistically significant (resp., *p* = 0.423, *p* = 0.128, and *p* = 0.423). In the diabetic sham group, the BUN, Cr, and NGAL values were found to be significantly low compared to the diabetic IR + LIPC group (resp., *p* = 0.037, *p* = 0.016, and *p* = 0.006). In the diabetic IR + LIPC group, though all 3 values were higher than the diabetic IR group, this difference was not statistically significant (resp., *p* = 0.423, *p* = 0.065, and *p* = 0.201). Biochemical data of the groups are presented in Table 3.

## 4. Discussion

This study found that histomorphological assessment results in diabetic rats showed that LIPC did not reduce or have a protective effect against IR injury. In fact, in addition to not having a protective effect, in the LIPC group, the histological score values for renal mononuclear cell infiltration, capillary vasodilatation, and structural changes in proximal tubules were higher than the values in the other groups, leading to the consideration that it had negative effects on renal IR injury. Biochemically the 3 biomarkers of BUN, Cr, and NGAL in diabetic rats showed that LIPC had no protective effect on renal IR injury.

Diabetes mellitus (DM) is a common and increasing chronic metabolic disease characterized by hyperglycemia, dyslipidemia, glycosuria, and metabolic disorders [6]. In diabetic kidneys initially diffuse and later exudative lesions develop. Hyalinization occurs in arterioles. Hyalinization in efferent arterioles forms histopathological lesions unique to diabetes. In the diabetic process, apart from diffuse and nodular intercapillary glomerulosclerosis (Kimmelstiel-Wilson syndrome), renal involvement may be observed linked to renal papilla necrosis, chronic pyelonephritis, atherosclerotic renal artery stenosis, and toxic nephropathy. Diabetic nephropathy is reported to increase linked to the increase in incidence of type II diabetes mellitus (DM). Though glomerular lesions are the best defined change in diabetic nephropathy, tubular atrophy, interstitial fibrosis, and inflammatory cell infiltration are the most significant characteristics of this disease. Diabetic nephropathy is a significant cause of mortality in diabetic patients. In Europe and America, 30–50% of type I diabetic patients and 5–15% of type II diabetic patients develop diabetic nephropathy. Patients with diabetic nephropathy present with glomerular hypertension and hyperperfusion and are more susceptible to I/R-induced renal injury. With the occurrence of DM-induced vessel lesions, renal tolerance to I/R is significantly compromised and the kidney is more likely to develop acute renal failure [16]. Many studies have previously reported an increased sensitivity to renal ischemia/reperfusion injury in DM rats. In these studies of the diabetic rat model induced with streptozotocin, 30 minutes of IR injury was shown to induce irreversible progressive renal damage. In diabetic rats this is characterized by interstitial fibrosis, inflammation, and tubular atrophy [17–19]. The mechanisms underlying enhanced vulnerability of the kidney to I/R injury in diabetes are not fully elucidated [17]. Thus, preventing renal I/R injury in DM by investigating possible protective strategies is clinically important.

Abu-Saleh et al. [20] in a diabetic rat model induced with streptozotocin identified histological changes in both diabetic and nondiabetic rats after 30 minutes of renal ischemia. They reported a broader pattern of injury in the diabetic ischemic kidney noted in the inner stripe of the outer medulla, including also congestion and inflammation, with all pathological parameters higher than 2.5 in morphological score. In our study in both the diabetic control group and the IR diabetic group the histomorphological scores for proximal tubules, mononuclear cell infiltration, and capillary vasodilation were higher than in the nondiabetic group. These different results compared to the study by Mejía-Vilet et al. [6] may be related to the diabetic monitoring duration of rats. In the study by Mejía-Vilet et al. rats were diabetically monitored for 5 days, while in our study this duration was 4 weeks. In our study all histomorphological scores in the diabetic control group were not statistically significant and were lower than in the diabetic IR group. This shows the negative effects expected in ischemia-reperfusion injury. Fouad et al. [17], with the same diabetic monitoring duration as our study, evaluated IR injury in nondiabetic and diabetic rats and showed dilatation especially of proximal tubules, vacuolar degeneration, and widespread necrosis in the diabetic IR group especially. The results of another study with two-week diabetic rats reported that the diabetic IR group had higher histomorphological scores compared to the diabetic control group [16]. Some of these studies aimed to better understand the mechanisms of renal ischemia injury while some researched the reducing and protective effect of a variety of agents on diabetes and IR injury. However, no study investigated the effects of mechanical ischemic preconditioning like LIPC, as in our study.

Mıcılı et al. [21] evaluated the therapeutic effects of LA administration histomorphometrically, ultrastructurally, and biochemically on renal damage formed as a result of hypertension and diabetes models together and separately in rats. They showed interstitial fibrosis, glomerulosclerosis index, mesangial matrix proliferation, and renal fibrosis increased significantly in diabetic and hypertensive rats, whereas in lipoic acid treatment groups these parameters were found to decrease significantly. There was a significant increase in the biochemical values and histomorphometric findings of this study in diabetic, 5/6 nephrectomy versus 5/6 nephrectomy + diabetic groups. In this study findings from all histological investigations, like proximal tubules, mononuclear cell infiltration, capillary vasodilation, and total cell injury score, in the diabetic rat groups were found to be significantly high compared to nondiabetic rats.

IR, where blood flow begins again after ischemia due to medication or mechanical intervention in ischemic tissues, paradoxically increases the injury caused by ischemia and causes greater injury to ischemic tissue than the injury caused by ischemia [22, 23]. Clinically IR injury is frequently observed after transplantation, stroke, myocardial infarction, shock/resuscitation, tourniquet applications, and extracorporeal shock-wave lithotomy [24]. Ischemic preconditioning is a method applied mechanically or pharmacologically prior to target organ ischemia to reduce the level of subsequent IR injury. Ischemic tolerance is induced by regulation of endothelial function, blood flow, and decreased macrophage as well as neutrophilic activity. This results in decreased endothelial injury and eventually decreased parenchymal injury. The aim in ischemic preconditioning is to apply ischemia and reperfusion to target organs in short intervals, to ensure that the target organs can tolerate ischemia well. High energy demands and the intense microvascular network of the kidneys make them vulnerable to IR injury, which is considered a major cause of kidney damage in renal artery stenosis and renal microvascular surgery. Wever et al. [25] in a meta-analysis of IPC studies in animal kidneys found many animal studies evaluating the protective effects of LIPC and RIPC on renal IRI using different stimuli and methods. The results of this meta-analysis stated there was no consensus on how long the ischemia stimulus should be applied and what the ischemia duration and reperfusion intervals should be. They emphasized that there was still no full explanation of the role played by age, sex, genetics, and comorbidities. Our results are one of the first studies evaluating the efficacy of LIPC on diabetic renal IRI. We propose that future clinical studies should be designed to optimize IPC efficacy for certain patient groups and that animal studies in this area can inform the design of such clinical trials. Furthermore, a better mechanistic insight is needed for the cause of the observed interspecies difference. Limiting factors are that oxidative stress and inflammatory mediators which are also responsible for IR injury and the neurogenic pathway were not examined in the study.

Renal IR injury can be induced using two different methods in experimental animal models. One of these is contralateral renal artery or renal pedicle clamping and unilateral nephrectomy while the other is bilateral renal pedicle or renal artery clamping [17, 26, 27]. In addition the duration of renal ischemia is very important and generally is limited to 30–60 minutes. Renal ischemia duration of more than sixty minutes may cause acute tubular necrosis and renal failure. With renal ischemia durations of less than 30 minutes, rapid proliferation of tubular epithelial cells may repair damaged renal tubules and this may be accompanied by improved renal functions [17, 26, 27]. Forty-five minutes of renal ischemia duration may cause findings of IR injury. Additionally, this injury may include renal failure linked to tubular epithelial cell proliferation with irreversible acute tubular necrosis. In previous studies, renal vessels were noninvasively clamped to induce ischemia for 45 min followed by reperfusion for 4, 6, 12, and 24 h [28]. Thus, in the present study, bilateral renal pedicles were clamped to induce ischemia for 45 min followed by reperfusion for 4 h.

Local ischemic preconditioning has been investigated as a surgical tool for many years [29]. Although LIPC does reduce reperfusion injury [28] as well as its systemic consequences [30], in a literature search, we found no study that evaluated the effects of LIPC for renal IR injury in a diabetic rat model. This study showed that LIPC treatment could not provide significant nephroprotective effects in streptozotocin-induced diabetic rats exposed to renal I/R.

In a review, McCafferty et al. [31] debated the relationship and possible mechanisms between ischemic conditioning in animal models and accompanying comorbidities. The results of this review found that comorbidities such as diabetes, hypertension, hypercholesterolemia, kidney disease, multiple medication use, and cerebrovascular disease reduced the clinical efficacy of ischemic preconditioning strategies. In the DM IR group, all histopathological scores were found to increase, though not significantly, compared to the DM sham group. The total cell injury score value especially was close to significance. These findings suggest that renal I/R injury may have severely impaired renal function; thus, renal I/R injury was successfully induced in these rats. Our findings are similar to the study findings of Zhou et al. [16] evaluating the protective effects of sevoflurane pretreatment of diabetic rats. Additionally when our biochemical results are compared with the diabetic sham, IR diabetic, and nondiabetic rats in the Zhou study, the clearly high serum Cr and BUN levels are similar. However, according to our results when the diabetic sham group and diabetic IR group are compared, the increase in serum BUN, Cr, and NGAL levels was not statistically significant. There was a clear significant increase histopathologically and biochemically between the diabetic sham and diabetic IR + LIPC groups. This leads to the consideration that LIPC application additionally increased ischemic damage in diabetic kidneys.

Many studies in recent times, especially on acute kidney injury, have proven the importance of the early diagnostic biomarker of NGAL, a protein with monomeric structure [32, 33] first determined as a protein linked to neutrophil gelatinase [25]. Neutrophil gelatinase-associated lipocalin was initially characterized as found in neutrophil lysosomes but later was observed to be expressed in a variety of tissues such as renal tubular epithelium, colon, prostate, and breast tissue [34]. NGAL production rapidly increases in response to renal epithelium damage or inflammation. After acute kidney injury, NGAL protein is easily identified in blood and urine. Experimentally after acute tubular injury mRNA of NGAL in the kidney increases 1000 times, with 10-fold increase in plasma and 100-fold increase in urine shown with acute kidney injury using the Western-blotting technique [34].

In our study we identified that serum NGAL levels were higher in diabetic rats compared to nondiabetic rats. However there was no significant difference found between the serum NGAL levels in the diabetic sham group and diabetic IR group. In diabetic kidneys after IR injury, higher serum NGAL levels were identified; however this difference was not statistically significant. One potential drawback to NGAL is the considerable extrarenal production that can be seen in states of systemic stress, even in the absence of renal damage; it has also been noted to increase in states of chronic, not just acute, kidney dysfunction. Nevertheless, in a large meta-analysis, NGAL did have prognostic significance for clinical outcomes, notably the need for dialysis and also mortality [11].

In conclusion, LPIC application did not reduce renal IR injury in the histomorphological and biochemical parameters of diabetic rats. As a result to better understand the physiopathological changes caused by diabetes and develop multidisciplinary approaches for preventive strategies, more advanced studies to determine underlying mechanisms are required. This data will provide clues about whether translation to humans is feasible. As LIPC has no effect in preventing diabetic IRI, in fact causing more harm, we believe research is required into other methods such as remote ischemic preconditioning and pharmacological agents.

## Figures and Tables

**Table 1:** Glucose values in rats.

Blood glucose	Basal	Day 3	1st week	2nd week	4th week	*p* value
Nondiabetic rats	109,1	109,3	107,5	111,6	110,9	<0.005
Diabetic rats	105,3	373,8	309,4	325,8	392,6	

*p* value: Friedman repeated measures.

**Table 2:** Histopathologic scores in groups.

Groups	Proximal tubulus	Mononuclear cell infiltration	Capillary vasodilation	Total cell injury score
Group I (nondiabetic sham) (*n* = 7)	0.14 ± 0.38 0.0 0-1	0.29 ± 0.49 0.0 0-1	0.29 ± 0.49 0.0 0-1	0.71 ± 1.11 0.0 0–3

Group II (diabetic sham) (*n* = 6)	1.33 ± 0.52 1.0 1-2	1.33 ± 0.52 1.0 1-2	1.17 ± 0.41 1.0 1-2	3.83 ± 0.98 3.5 3–5

Group III (diabetic IR) (*n* = 6)	1.83 ± 0.41 2.0 1-2	1.66 ± 0.52 2.0 1-2	1.5 ± 0.55 1.5 1-2	5.0 ± 1.09 5.0 4–6

Group IV (diabetic IR + LIPC) (*n* = 6)	2.33 ± 0.52 2.0 2-3	2.17 ± 0.75 2.0 1–3	2.17 ± 0.41 2.0 2-3	6.67 ± 0.82 6.5 6–8

*p values *				
*p*_12_	0.003	0.008	0.010	0.004
*p*_13_	0.001	0.004	0.006	0.002
*p*_14_	0.001	0.003	0.001	0.002
*p*_23_	0.093	0.269	0.241	0.082
*p*_24_	0.014	0.057	0.006	0.003
*p*_34_	0.092	0.206	0.043	0.020

Group I: nondiabetic sham, Group II: diabetic sham, Group III (diabetic IR): renal ischemia/reperfusion injury in diabetic rats group, and Group IV (diabetic IR + LIPC): renal ischemia/reperfusion injury in diabetic rats group and local ischemia preconditioning; Mann-Whitney *U* test was conducted to compare two independent groups. Values are mean ± 1 SD, median (minimum-maximum).

*p*_12_: comparison of nondiabetic sham and diabetic sham.

*p*_13_: comparison of nondiabetic sham and diabetic IR.

*p*_14_: comparison of nondiabetic sham and diabetic IR + LIPC.

*p*_23_: comparison of diabetic sham and diabetic IR.

*p*_24_: comparison of diabetic sham and diabetic IR + LIPC.

*p*_34_: comparison of diabetic IR and diabetic IR + LIPC.

**Table 3:** Biochemical data of the groups.

Groups	BUN	Cr	NGAL
Group I (nondiabetic sham) (*n* = 7)	49.83 ± 21.80 44.20 31.90–95.60	0.46 ± 0.18 0.44 0.27–0.80	446.43 ± 179.17 386.00 314.00–839.00

Group II (diabetic sham) (*n* = 6)	68.52 ± 15.07 72.20 39.30–83.50	0.43 ± 0.21 0.39 0.24–0.82	350.17 ± 35.62 352.5 295.00–395.00

Group III (diabetic IR) (*n* = 6)	79.62 ± 14.93 78.20 62.10–101.70	0.62 ± 0.20 0.66 0.29–0.83	891.17 ± 1308.03 363.50 296.00–3560.00

Group IV (diabetic IR + LIPC) (*n* = 6)	84.68 ± 16.79 89.75 52.00–96.80	0.81 ± 0.80 0.81 0.69–0.91	1107.40 ± 1294.21 412 397.00–3385.00

*p values *			
*p*_12_	0.086	0.567	0.153
*p*_13_	0.022	0.153	0.568
*p*_14_	0.032	0.010	0.167
*p*_23_	0.423	0.128	0.423
*p*_24_	0.037	0.016	0.006
*p*_34_	0.423	0.065	0.201

Group I: nondiabetic sham, Group II: diabetic sham, Group III (diabetic IR): renal ischemia/reperfusion injury in diabetic rats group, Group IV (diabetic IR + DIPC): renal ischemia/reperfusion injury in diabetic rats group and local ischemia preconditioning, BUN: blood urea nitrogen, and serum NGAL: Cr: blood creatinine level, Mann-Whitney *U* test was conducted to compare two independent groups. Values are mean ± 1 SD, median, minimum, and maximum.

*p*_12_: comparison of nondiabetic sham and diabetic sham.

*p*_13_: comparison of nondiabetic sham and diabetic IR.

*p*_14_: comparison of nondiabetic sham and diabetic IR + LIPC.

*p*_23_: comparison of diabetic sham and diabetic IR.

*p*_24_: comparison of diabetic sham and diabetic IR + LIPC.

*p*_34_: comparison of diabetic IR and diabetic IR + LIPC.
